# Circadian Clock Programming of Anticipatory Antiviral Immunity Gates Enteric Virus Infection Susceptibility

**DOI:** 10.64898/2026.05.15.725500

**Published:** 2026-05-16

**Authors:** Temitope O. Oshinowo, Robert W. Maples, Mikal A. Woods Acevedo, Broc T. McCune, Hunter Dalton, Ishea Johnson, Devin A. Simpkins, Urbashi Basu, Chaitanya Dende, Vera L. Tarakanova, Julie K. Pfeiffer, John F. Brooks

**Affiliations:** 1 Department of Molecular Biology, Princeton University; Princeton NJ, 08540, USA.; 2 Department of Microbiology, University of Texas Southwestern Medical Center; Dallas TX, 75390, USA.; 3 Department of Immunology, University of Texas Southwestern Medical Center; Dallas TX, 75390, USA.; 4 Department of Microbiology and Immunology and Cancer Center, Medical College of Wisconsin, Milwaukee, Wisconsin, USA.

## Abstract

Susceptibility to viral infection varies widely but is not fully explained by genetics, immune status, or exposure level. We show that time of day strongly influences infection outcome, with up to 100-fold differences in enteric viral burden depending on infection timing. This temporal gating is abolished in mice lacking a functional circadian clock. We identify the antiviral transcription factor IRF1 as a direct target of the circadian transcription factor BMAL1, resulting in rhythmic expression of a basal antiviral gene program prior to infection. Loss of IRF1 eliminates this program and abrogates time-of-day–dependent differences in viral replication. This circuit operates within intestinal myeloid cells, establishing a preexisting antiviral state. These findings indicate that the circadian clock programs host susceptibility in the intestine, before infection occurs.

## INTRODUCTION

Susceptibility to viral infection varies widely among individuals, yet the determinants of this variability remain incompletely understood. Host genetics, immune status, and route/level of exposure all shape infection outcomes, but these factors alone do not fully account for observed differences in viral burden. One fundamental dimension of host biology that has received little attention is timing of infection during the day-night cycle.

The circadian clock is an evolutionarily conserved system that synchronizes physiology with the day-night cycle. By coordinating gene expression, metabolism, and behavior (1, 2) the clock enables organisms to anticipate predictable environmental changes rather than merely react to them. Immunologically, circadian regulation influences leukocyte trafficking (3–6), cytokine production (3), and inflammatory responses (7–11). These studies have largely focused on how the clock modulates the magnitude or timing of immune responses after stimulation. Prior studies have shown that circadian timing can influence infection with respiratory and systemic viruses (12–16). In these settings, time-of-day effects have been attributed largely to variation in host responses after infection or to virus–host interactions during systemic spread. Whether circadian programs instead establish a preexisting antiviral state that determines susceptibility, particularly in the intestine, remains unclear.

Enteric viral infections provide a powerful context for examining temporal regulation of host defense. In addition to rhythmic food intake, the intestinal mucosa is constantly exposed to environmental antigens and microbial products, requiring strategies that balance nutrient acquisition, metabolism, and immune protection vs. damage (17). Mammalian enteric virus infections are common, and outcomes range from mild symptoms to fatal diarrhea or systemic disease. Innate antiviral defenses in this setting rely on basal expression of viral sensors and restriction factors that act early in infection (18, 19), often before robust interferon amplification occurs. This raises the possibility that temporal regulation at mucosal surfaces may operate through anticipatory immune programs distinct from those described in systemic or respiratory infections. How these basal antiviral programs are regulated in time, and whether they influence infection outcome, is not well understood.

Here, we investigated whether host susceptibility to enteric viral infection is temporally regulated across the day-night cycle. In mice perorally inoculated with the model enteric picornavirus coxsackievirus B3 (CVB3), we show that infection outcome depends strongly on the time of exposure, revealing daily windows of host resistance and susceptibility. We identify a circadian clock-controlled immune program that operates within myeloid cells of the intestinal lamina propria to regulate IRF1 expression and downstream antiviral gene expression. Disruption of this program abolishes temporal differences in viral susceptibility without altering circadian behavior or feeding rhythms. Together, these findings demonstrate that the circadian clock does not merely tune immune responses after infection but instead establishes a temporally gated and anticipatory antiviral state-a “head start” on innate responses during the rest phase in nocturnal animals. By defining time as a critical axis of host susceptibility, this work reframes clock regulation as a fundamental component of anticipatory antiviral defense.

## RESULTS

### Time of infection determines enteric virus replication efficiency

**Fig. 1.**
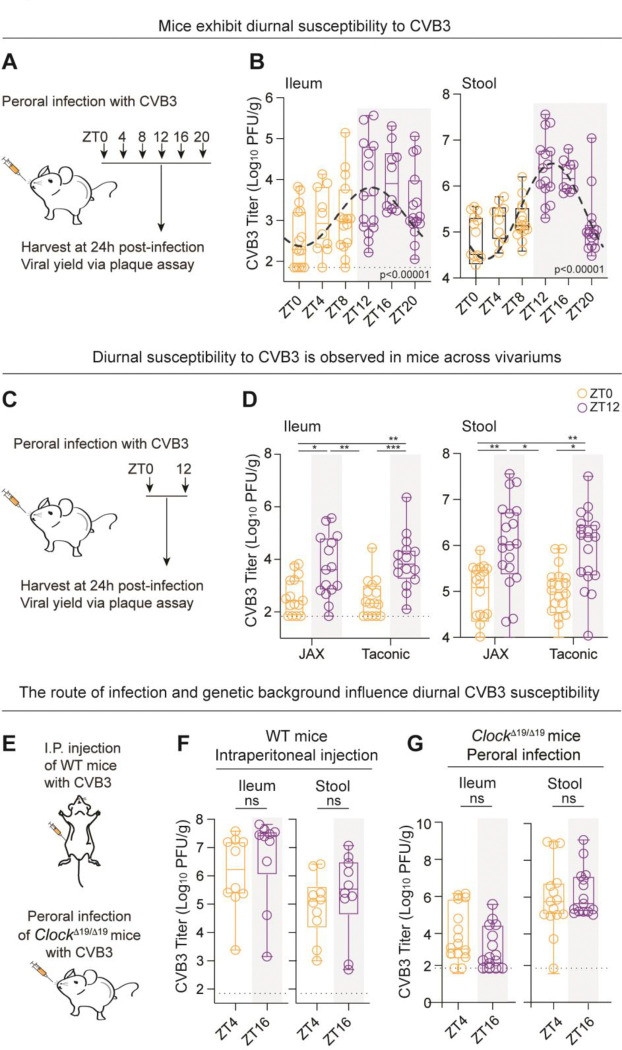
Time of day determines host susceptibility to enteric viral infection. **(A)** Mice were perorally infected with 10^9^ PFU CVB3 at six timepoints across the day-night cycle and tissues were harvested at 24h post-infection followed by plaque assay using HeLa cells. **(B)** Viral load in the ileum and stool of wildtype mice at 24h post-infection. Black dashed line shows rhythmicity analysis (P < 0.0001) of peak and trough within the data set, and dotted line indicates limit of detection. **(C)** Wildtype C57BL/6 mice from The Jackson Laboratory (JAX) versus Taconic Biosciences were perorally infected with 10^9^ PFU CVB3 at one timepoint during the day (ZT0) and one timepoint during the evening (ZT12), followed by viral titer analysis at 24h post-infection. **(D)** Viral load in the ileum and stool of Jackson (JAX) vs. Taconic mice at 24h post-infection. **(E)** Wildtype C57BL/6 mice were intraperitoneally injected with 10^5^ PFU CVB3 at ZT4 or 16 (top) or mice with a disrupted circadian clock (*Clock*^*Δ19/Δ19*^*)* were perorally infected with 10^9^ PFU CVB3 at ZT4 or 16 (bottom), and viral load was determined by plaque assay at 24h post-infection. **(F)** Viral load in the ileum and stool of intraperitoneally injected wildtype mice at 24h post-infection. **(G)** Viral load in the ileum and stool of perorally infected *Clock*^*Δ19/Δ19*^ mice at 24h post-infection. PFU, plaque forming units; CVB3, coxsackievirus B3; ZT4, zeitgeber time 4 (10:00 AM); ZT16, zeitgeber time 16 (10:00 PM), *Clock*, circadian locomotor output cycles kaput. Means ± SEM are plotted; *P < 0.05, **P < 0.01; ***P < 0.001; ****P < 0.0001 as determined by Student’s t-test or ANOVA. ns, not significant. Black dashed line indicates rhythmicity ****P <0.0001, generated with CircaCompare.

To test whether host susceptibility to enteric viral infection varies by time of day, we perorally infected mice with coxsackievirus B3 (CVB3) at 4-hour intervals over a 24-hour period beginning at zeitgeber time (ZT) 0 (6 am, lights on) (Fig. 1A). Viral loads were quantified by plaque assays 24 hours after infection. Viral loads exhibited a pronounced dependence on infection timing: mice infected during the early light phase (ZT0 and ZT4) had significantly lower viral titers compared to mice infected during the dark phase, which begins at ZT12 (lights off). This temporal difference was evident throughout the gastrointestinal tract, with viral titers in the small intestine, cecum, and stool exhibiting diurnal oscillations (p < 0.0001) that varied by up to 116-fold depending on infection time (Fig. 1B, Fig. S1A, B). Given that enteric viruses spread by the fecal-oral route, elevated viral titers in stool may influence transmission efficiency.

Next, we examined several factors that may contribute to diurnal variation of infection efficiency: Intestinal microbiota, inoculation route, and a host transcription factor that establishes rhythmic gene expression. First, we determined whether variation in intestinal microbiota influences diurnal effects on CVB3 susceptibility. Our past work demonstrated that microbiota promote enteric virus infection (20–22), indicating that microbial communities can strongly influence viral outcomes in the gut. In addition, specific taxa, including segmented filamentous bacteria (SFB) that vary between animal facilities, diurnally modulate host immune pathways and protect against select enteric viruses (17, 23). Therefore, we sought to determine whether the observed temporal variation in CVB3 susceptibility was robust across distinct microbiota compositions. To address this, we compared infection in mice obtained from two commercial vendors that harbor distinct and well-characterized microbial communities, including the presence of SFB (Fig. 1C). Time-of-day-dependent differences in CVB3 replication efficiency were preserved in mice across vendors (Fig. 1D, Fig. S1C), suggesting that temporal variation in viral load is largely independent of microbial composition (17). Second, we determined whether diurnal effects on CVB3 replication were specific to the natural oral infection route (Fig. 1E). Mice infected via intraperitoneal injection that bypasses the intestinal tract failed to exhibit time-of-day–dependent differences in viral titers (Fig. 1F, Fig. S1D), indicating that temporal gating of viral susceptibility is specific to mucosal infection and is not observed following systemic inoculation. Finally, we examined if the core circadian transcription factor Circadian Locomotor Output Cycles Protein Kaput (CLOCK) influences viral loads across the day-night cycle (Fig. 1E). Mice with a dominant-negative mutation due to the deletion of the CLOCK exon 19, *Clock*^*Δ19/Δ19*^ mice, no longer exhibited time-of-day–dependent differences in viral load following peroral CVB3 infection (Fig. 1G, Fig. S1E). Together, these data demonstrate that host permissiveness to enteric viral infection is temporally regulated, conserved across distinct microbiomes, specific to the oral infection route, and requires an intact circadian clock.

### The circadian clock establishes rhythmic expression of the antiviral regulator IRF1

**Fig. 2.**
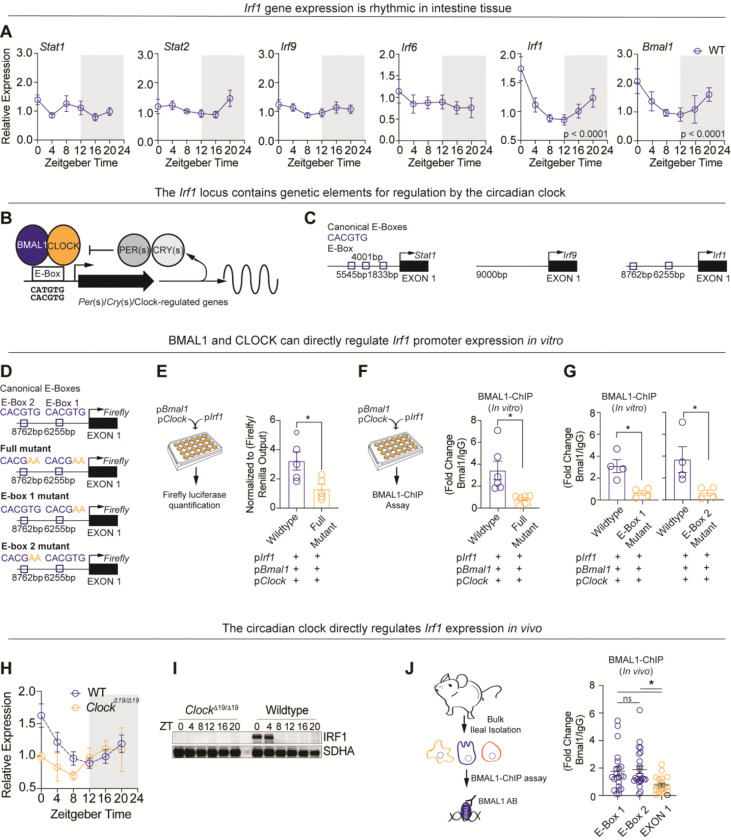
The circadian clock establishes rhythmic expression of the antiviral regulator IRF1. **(A)** qRT-PCR quantification of *Stat1, Stat2,* and select IRFs at six timepoints across the day-night cycle. *Irf1* and *Bmal1* are significantly rhythmic. **(B)** BMAL1 and CLOCK bind E-box elements in promoters to promote transcription of circadian-controlled genes, including their own repressor proteins, PERs and CRYs. **(C)** Putative E-box elements upstream of *Stat1, Irf9,* and *Irf1*. **(D)** Visualization of the *Irf1* promoter showing wildtype E-Boxes, and mutated (Δ) E-Boxes in luciferase reporters. **(E)** Left: Transfection-based luciferase reporter assay. Right: Quantification of *Irf1* promoter activity using a luciferase reporter assay for wildtype vs. mutated E-box elements. **(F)** Left: Transfection-based BMAL1 ChIP assay. Right: Quantification of BMAL1 binding to the *Irf1* promoter *in vitro* for wildtype vs. dual mutated E-box elements. **(G)** Quantification of BMAL1 binding to the *Irf1* promoter *in vitro* for wildtype vs. singly mutated E-box elements (E-box 1 vs. E-box 2). **(H)** qRT-PCR quantification of *Irf1* in wildtype mice versus *Clock* ^*Δ19/Δ19*^ mice at six timepoints across the day-night cycle. **(I)** Western blot examination of IRF1in wildtype mice versus *Clock* ^*Δ19/Δ19*^ mice. **(J)** Quantification of BMAL1 binding to the *Irf1* locus *in vivo.* Small intestine ileal tissue was harvested from wildtype mice at ZT0, followed by BMAL1-ChIP assay targeting E-box 1, E-Box 2 or a downstream control region, EXON1. Data are presented as the ratio of BMAL1 to control IgG antibody. *Stat*, signal transducer and activator of transcription; *Irf*, interferon regulatory factor; BMAL1, brain and muscle ARNT-like 1; *pIrf1, Irf1* plasmid (cloned); *pBmal1, Bmal1* plasmid (constitutively expressed); *pClock, Clock plasmid* (constitutively expressed). Means ± SEM are plotted; *P < 0.05, **P < 0.01; ***P < 0.001; ****P < 0.0001 as determined by Student’s t-test or ANOVA. ns, not significant.

To identify host immune regulators that could mediate time-of-day–dependent susceptibility, we examined the expression of key transcription factors involved in antiviral immunity at early time points after infection. Among multiple candidates, including *Stat1*, *Stat2*, and several interferon regulatory factors (IRFs), only *Irf1* exhibited robust circadian oscillation in expression (Fig. 2A, Fig. S2A–B). IRF1 is a central transcriptional regulator of antiviral immunity (24, 25). Unlike inducible interferon responses that are rapidly engaged following infection, IRF1 contributes to basal expression of antiviral genes, including sensors and effectors that restrict viral replication at initial stages (26, 27). IRF1 activity restricts diverse viral pathogens (28), yet how its expression is regulated under homeostatic conditions *in vivo* remains poorly defined. We found that *Irf1* expression peaked during the light phase and reached its nadir during the dark phase, coinciding with periods of maximal and minimal host resistance, respectively (Fig. 2A, Fig. 1B).

Next, we examined whether *Irf1* expression was directly regulated by circadian clock transcription factors. The core circadian clock operates through a transcriptional-translational feedback loop in which CLOCK and Brain and Muscle ARNT-Like 1 (BMAL1) transcription factors form a heterodimer that binds enhancer (E)-box motifs to regulate gene expression (Fig. 2B). Promoter analysis revealed that *Irf1* contains two conserved E-box elements proximal to its transcriptional start site (Fig. 2C). To test whether *Irf1* is a direct clock-controlled gene, we used luciferase reporter assays and chromatin immunoprecipitation (ChIP) *in vitro* and expression analysis plus ChIP *in vivo*.

For *in vitro* assays, we cloned the *Irf1* promoter upstream of a luciferase reporter and mutated one or both putative E-boxes (Fig. 2D). First, we assessed transcriptional activation in the presence of CLOCK and BMAL1 using luciferase reporter assays in transfected HEK293 cells (Fig. 2E). CLOCK and BMAL1 robustly activated the wildtype *Irf1* promoter, whereas mutation of the E-box elements abolished this effect (Fig. 2E). Next, we assessed binding of BMAL1 to the *Irf1* promoter using ChIP assays. We transfected HEK293 cells with *Irf1* promoter plasmids and CLOCK and BMAL1 expression plasmids, isolated chromatin, immunoprecipitated with anti-BMAL1 or control antibody, and quantified bound DNA using qPCR for the *Irf1* promoter region (Fig. 2F). We found BMAL1-specific binding to the wildtype *Irf1* promoter, but reduced binding to *Irf1* promoter with mutated E-boxes (Fig. 2F–G).

For *in vivo* assays, we examined *Irf1* expression in ileal tissues, and we performed ChIP to assess BMAL1 binding to the *Irf1* promoter. As measured by qRT-PCR, *Irf1* RNA was oscillatory over time in wildtype mice but not in *Clock*^*Δ19/Δ19*^ mice (Fig. 2H). At the protein level, IRF1 levels were high in wildtype mice at ZT0/4 but low at other time points in wildtype mice and at all time points in *Clock*^*Δ19/Δ19*^ mice (Fig. 2I). Furthermore, in *Clock*^*Δ19/Δ19*^ mice, *Irf1* expression was arrhythmic (Fig. S2C). ChIP assays performed with intestinal tissue further demonstrated BMAL1 binding to both E-boxes at the endogenous *Irf1* locus *in vivo,* which was significantly higher than binding to control downstream sequence (EXON1)(Fig. 2J). These data establish *Irf1* as a direct transcriptional target of the circadian clock proteins CLOCK and BMAL1.

### IRF1 is required for temporal gating of antiviral gene expression

**Fig. 3:**
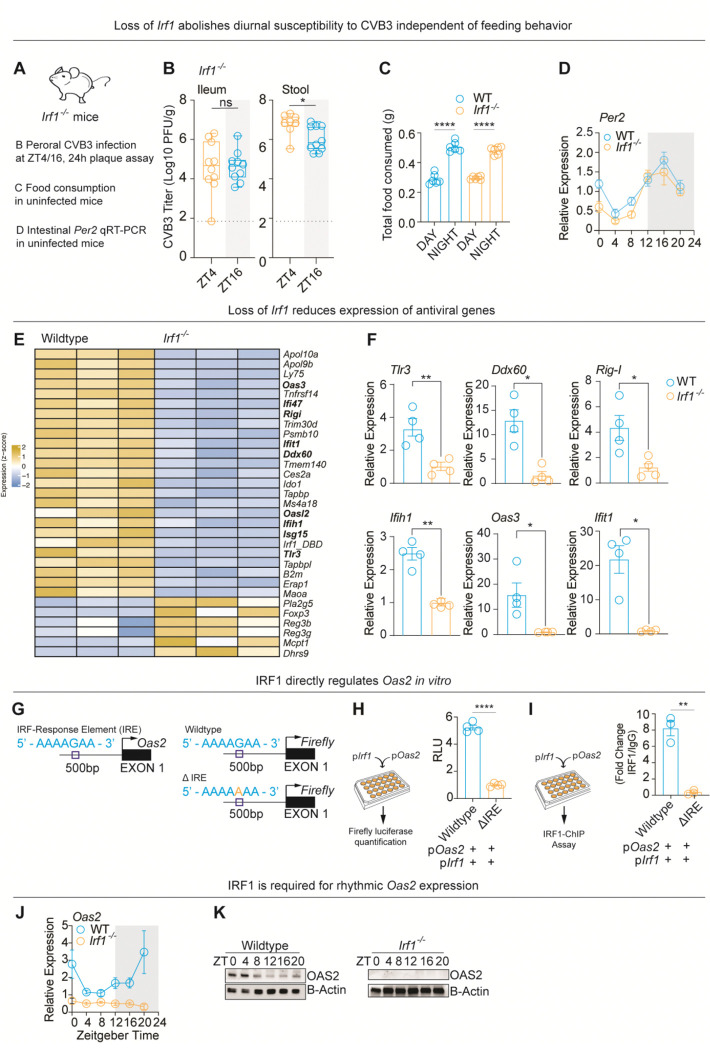
IRF1 is required for temporal gating of antiviral immunity. **(A)** Characterization of *Irf1*^−/−^ mice, including peroral infection and titer assay, food consumption quantification, and assessment of circadian gene expression. **(B)** *Irf1*^−/−^ mice were perorally infected with 10^9^ PFU CVB3 at one timepoint during the day (ZT4) and one timepoint during the evening (ZT16) and tissues were harvested at 24h post-infection followed by plaque assay using HeLa cells. **(C)** Feeding rhythms were examined in wildtype vs. *Irf1*^−/−^ mice across several days. The total amount of food consumed per day and night was quantified. **(D)** qRT-PCR quantification of *Per2* at six timepoints across the day-night cycle in wildtype vs. *Irf1*^−/−^ mice. **(E)** Heatmap comparing gene expression in wildtype vs. *Irf1*^−/−^ mice from RNA-seq of ZT0 ileum tissue. **(F)** ZT0 qRT-PCR quantification of *Tlr3*, *Ddx60*, *Rig-I*, *Ifih1*, *Oas3*, and *Ifit1* in wildtype versus *Irf1*^−/−^ mice. **(G)** Visualization of the *Oas2* promoter showing wildtype IRE, and mutated (Δ) IRE in luciferase reporters. **(H)** Left: Transfection-based *Oas2* luciferase reporter assay. Right: Quantification of *Oas2* promoter activity using a luciferase reporter assay for wildtype vs. mutated IRE. **(I)** Left: Transfection-based IRF1 ChIP assay. Right: Quantification of IRF1 binding to the *Oas2* promoter *in vitro* for wildtype vs. mutated IRE. **(J)** qRT-PCR quantification of *Oas2* at six timepoints across the day-night cycle in wildtype and *Irf1*^−/−^ mice. **(K)** Western blot examination of OAS2 at six timepoints across the day-night cycle in wildtype and *Irf1*^−/−^ mice. *Per2, Period 2*. *Tlr3*, toll-like receptor 3; *Ddx60*, DExD/H-box helicase 60; *Rig-I*, retinoic acid-inducible gene I; *Ifih1*, interferon induced with helicase C domain 1; *Oas3*, 2'−5'-oligoadenylate synthetase 3; *Ifit1* interferon-induced protein with tetratricopeptide repeats 1; IRE, IRF response element; *Oas2* 2'−5'-oligoadenylate synthetase 2; *pOas2, Oas2* plasmid (cloned); *pIrf1, Irf1* plasmid (constitutively expressed). Means ± SEM are plotted; *P < 0.05, **P < 0.01; ***P < 0.001; ****P < 0.0001 as determined by Student’s t-test or ANOVA. ns, not significant.

To determine whether IRF1 is required for time-of-day–dependent resistance to infection, we examined *Irf1*^−/−^ mice (Fig. 3A, Fig. S3A–B). Loss of IRF1 abolished temporal differences in CVB3 replication, with viral titers remaining comparable regardless of infection time (Fig. 3B). Given that food intake influences oscillatory gene expression and knockout mice may have altered feeding behaviors, we quantified food intake in wildtype vs. *Irf1*^−/−^ mice. *Irf1*^−/−^ mice retained normal feeding rhythms (Fig. 3C, Fig. S3C) and intact oscillations of core clock genes (Fig. 3D, Fig. S3D), indicating that loss of temporal gating did not reflect disruption of circadian behavior or clock function.

Next, we evaluated the antiviral programs downstream of IRF1. First, we performed RNA sequencing from ileal tissue at ZT0, corresponding to peak IRF1 expression in wildtype animals. IRF1-deficiency resulted in reduced expression of multiple genes involved in viral sensing and restriction, including *Oas2*, *Oas3*, *Oasl2*, *Ddx60, Ddx58* (RIG-I), *Ifih1* (MDA5), *Ifit1*, and *Tlr3* (Fig. 3E), indicating that IRF1 sustains a basal antiviral transcriptional state during periods of maximal resistance. Using qRT-PCR, we confirmed decreased expression of these and other immune genes when comparing *Irf1*-deficient mice to wildtype mice (Fig. 3F).

IRF1 binds to IRF-Response Elements (IREs) in promoters of target genes and we used *Oas2* as a representative IRF1 target gene to gain more insight into rhythmic control of innate immune responses. We generated luciferase reporters with wildtype or mutated IREs in the *Oas2* promoter and used them for *in vitro* assays (Fig. 3G–I). IRF1 induced reporter activity from the wildtype promoter, but not from an IRE mutant (Fig. 3H). Next, we assessed binding of IRF1 to the *Oas2* promoter using ChIP assays in HEK293 cells (Fig. 3I). We found IRF1-specific binding to the wildtype *Oas2* promoter, but reduced binding to *Oas2* promoter with a mutated IRE (Fig. 3I). Finally, we quantified expression of *Oas2* in wildtype vs. *Irf1*^−/−^ mice. *Oas2* gene and protein expression was minimal in *Irf1*^−/−^ mice, but detectable and rhythmic in wildtype mice (Fig. 3J, K, Fig. S3F). Together, these data indicate that IRF1 is required to establish a temporally regulated antiviral state that restricts viral replication.

### A myeloid cell-intrinsic program controls temporal antiviral resistance

**Fig. 4:**
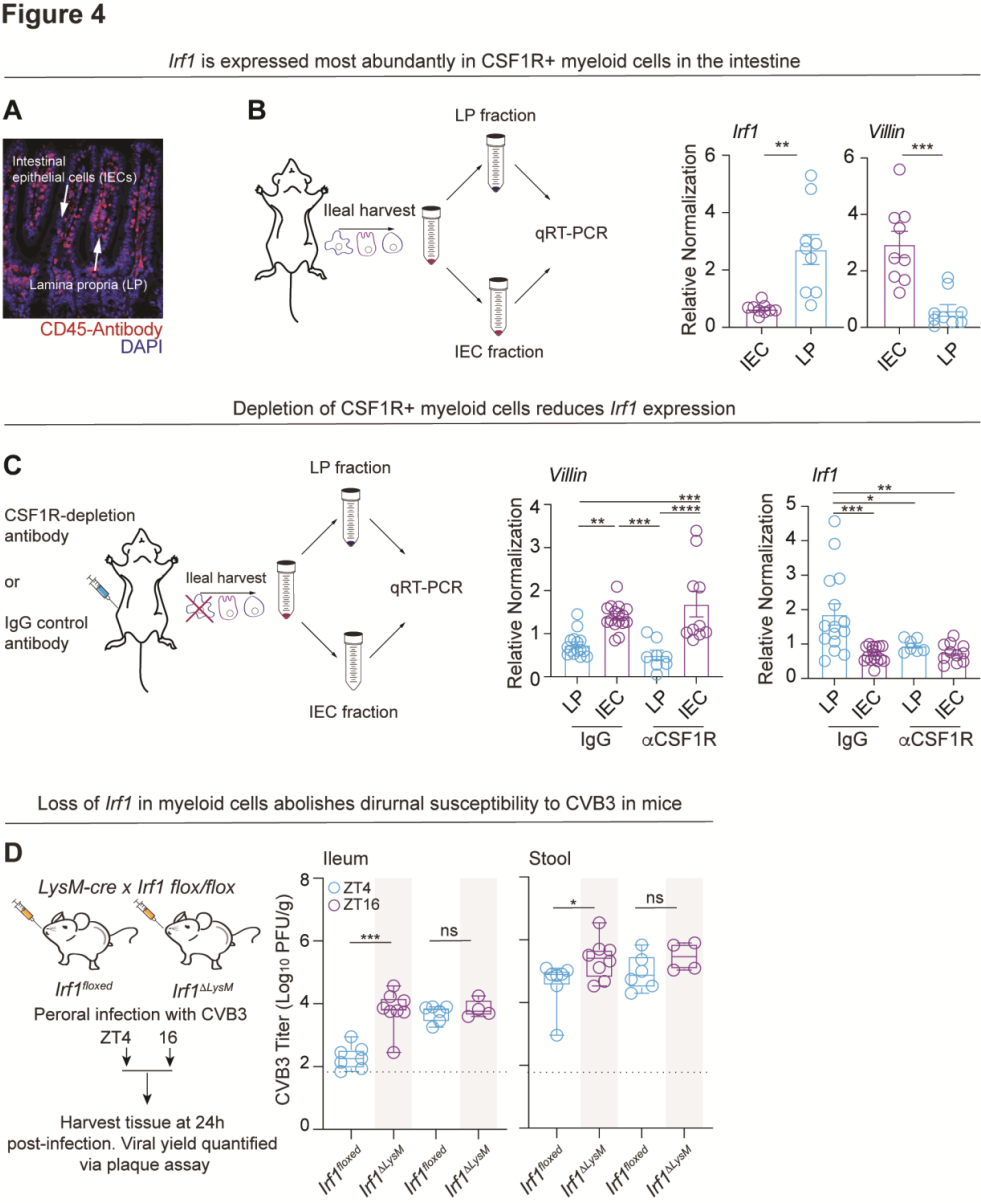
A myeloid cell–intrinsic program controls temporal antiviral resistance. **(A)** Microscopy of murine small intestine, showing intestinal epithelial cells (IECs) and lamina propria (LP) cells (stained with CD45 antibody) beneath. **(B)** Gene expression in IEC vs. LP. Small intestine from wildtype was collected at ZT4, IECs and LP cells were purified, and qRT-PCR quantified expression of *Irf1* and *Villin*, a marker of IECs. **(C)** Gene expression in mice with depleted CSF1R+ cells. Wildtype mice were injected at ZT4 with a macrophage depletion antibody (anti-CSF1R) or IgG control and 72h later intestine was collected, IECs and LP cells were isolated, and qRT-PCR quantified expression of *Irf1* and *Villin.* **(D)** Viral load in the ileum and stool of perorally infected mice deficient for *Irf1* specifically in myeloid cells (*Irf1*^*ΔLysM*^) and controls (*Irf1*^*floxed*^). Mice were infected with 10^9^ PFU CVB3 at one timepoint during the day (ZT0) and one timepoint during the evening (ZT12) followed by quantification of viral load by plaque assay. IECs, intestinal epithelial cells; LP, lamina propria; CSF1R Colony-stimulating factor 1 receptor; IgG, immunoglobulin G. Means ± SEM are plotted; *P < 0.05, **P < 0.01; ***P < 0.001; ****P < 0.0001 as determined by Student’s t-test or ANOVA. ns, not significant.

We next sought to identify the cellular basis of rhythmic IRF1-dependent antiviral effects. The intestine contains a variety of cell types, including epithelial cells that overlay a large number of immune and other cell types within the lamina propria (Fig. 4A). We first compared *Irf1* expression across intestinal compartments by isolating lamina propria cells vs. epithelial cells from uninfected wildtype mice at ZT0. *Irf1* expression was markedly enriched in lamina propria cells relative to epithelial cells (Fig. 4B). Myeloid cells are abundant (29) within the lamina propria and previous work established that *Irf1* is expressed in myeloid populations (30). Consistent with this, antibody-mediated depletion of CSF1R-positive myeloid cells at ZT4 significantly reduced *Irf1* expression within the lamina propria (Fig. 4C), identifying myeloid cells as a source of IRF1 in the intestinal immune compartment. Finally, we examined viral loads in mice with lineage-specific deletion of *Irf1* in myeloid cells (31), both to narrow the relevant cell types subject to these anticipatory antiviral pathways and to rule out confounding effects of global *Irf1* deletion including impacts on T cell development (27). Myeloid-specific loss of IRF1 in *Irf1*^*ΔLysM*^ mice, but not in *Irf1*^*fl/fl*^ controls, abolished time-of-day–dependent differences in CVB3 titers, phenocopying global *Irf1* deficiency (Fig. 4D). Together, these data demonstrate that myeloid cell-intrinsic IRF1 expression, driven by circadian transcription factors, establishes daily rhythms in antiviral programs.

## DISCUSSION

Our findings establish that host susceptibility to enteric viral infection oscillates by time of day through anticipatory antiviral responses. We identify a myeloid cell–intrinsic program that controls expression of the antiviral transcription factor IRF1, establishing daily rhythms in basal antiviral gene expression and defining temporal windows of host resistance. These results reveal time of infection as an underappreciated determinant of viral outcome, comparable to host genetics, immune status, or route of exposure, and highlight the importance of reporting time of day for reproducibility of infection studies. Notably, anticipatory immune regulation has also been described in plants, where circadian programs precondition defense pathways prior to pathogen exposure (32). Together, these findings suggest that anticipatory immunity may represent a conserved, cross-kingdom strategy aligning host defense with predictable environmental risk.

Our data suggest that clock control acts early in infection. Viral titers diverge within the first 24 hours, consistent with effects on the initial cycles of replication. Rhythmic IRF1 and downstream antiviral gene expression occur prior to infection, supporting a basal antiviral state rather than an inducible response. The IRF1-dependent gene set includes sensors and restriction factors such as MDA5(33), OAS family members (34) and TLR3 (35, 36), indicating coordinated regulation of early antiviral defenses. Notably, time-of-day differences are observed following oral but not systemic infection, indicating that temporal control operates at the mucosal interface during infection by the natural oral route. Together, these findings support a model in which timing shapes the earliest host–virus interactions, biasing infection outcome before widespread replication is established. This mode of regulation contrasts with prior clock-virus studies, which have largely focused on how time of day influences host responses after infection or effects on viral replication dynamics (12–16). Here, we show that the circadian clock instead programs antiviral immunity prior to infection, establishing a basal state that gates susceptibility at the moment of exposure.

Clock regulation of antiviral immunity operates through a cell-type–specific transcriptional circuit that establishes a transient antiviral state. We identify CSF1R-positive myeloid cells in the intestinal lamina propria as a major source of rhythmic IRF1 expression, positioning these cells as temporal sentinels that preemptively shape the antiviral landscape. Within this compartment, the circadian clock selectively targets IRF1 rather than broadly oscillating interferon pathways, focusing regulation on a single transcriptional node that sustains basal antiviral gene expression. This architecture may balance immune readiness with the metabolic and inflammatory costs of sustained activation, enabling anticipatory protection without continuous immune stimulation (37).

The emergence of a temporally gated basal antiviral state in mice raises the question of why such anticipatory immunity arose and is maintained. One possibility is that this program reflects evolutionary selection imposed by recurrent or predictable pathogen exposure at specific times of day (37). In this model, individuals with elevated antiviral defenses aligned with periods of increased exposure risk would have enhanced survival, leading to retention of anticipatory immunity. Limiting immune activation to defined temporal windows may optimize the tradeoff between protection and cost, minimizing metabolic burden and immunopathology while preserving effective host defense. In nocturnal animals such as mice, this anticipatory immune response may be coordinated with behavior and infection dynamics. Following oral exposure, likely during the active dark phase, productive viral replication in the intestine is delayed by several hours due to gastrointestinal transit (21), creating a temporal separation between exposure and replication. Subsequently, in the early light phase, peak antiviral gene expression aligns with inefficient viral replication. This temporal offset positions viral replication within a pre-existing antiviral state, allowing a clock driven antiviral program to restrict early viral replication.

In summary, our work reveals a rhythmic innate immune program that establishes antiviral resistance through IRF1, redefining host susceptibility as a dynamic and anticipatory state. This framework may help explain variability in infection outcomes not accounted for by exposure, genetics, or immunocompetence, and has implications for understanding viral transmission and optimizing the timing of interventions.

## Supplementary Material

Supplement 1

## Data Availability

Bulk RNA-sequencing data is available here: https://osf.io/te8am/overview?view_only=8193460b964c4379b679bc2d29c53952
