# Supplementary material for: Circadian Clock Programming of Anticipatory Antiviral Immunity Gates Enteric Virus Infection Susceptibility
Source: bioRxiv. 2026 May 16:2026.05.15.725500. Preprint. [Version 1] doi: 10.64898/2026.05.15.725500 (PMC13192825; doi:10.64898/2026.05.15.725500)
Supplement: Supplement 1 [file NIHPP2026.05.15.725500v1-supplement-1.pdf]

bioRxiv preprint doi: https://doi.org/10.64898/2026.05.15.725500; this version posted May 16, 2026. The copyright holder for this preprint (which was not certified by peer review) is the author/funder, who has granted bioRxiv a license to display the preprint in perpetuity. It is made available under a CC-BY 4.0 International license.

# Supplementary Materials

## Materials and Methods

### Virus and cells

HeLa cells were grown at 37°C in Dulbecco's Modified Eagle Medium (DMEM) media supplemented with 10% newborn calf serum (Fisher Scientific, #NC1928575) and Penicillin-Streptomycin (10,000U penicillin, 10mg streptomycin, Millipore Sigma, #P4333-100ml). A CVB3-H3 stock (a gift from Marco Vignuzzi; GenBank: U57056.1) was generated by co-transfecting an infectious clone plasmid with a T7 RNA polymerase expression plasmid into HeLa cells. Further amplification was performed to generate a high-titer viral stock. Plaque Forming Units (PFU) were quantified by plaque assay using HeLa cells *(38)*.

### Mice

All animal procedures followed NIH guidelines and were approved by the University of Texas Southwestern Medical Center IACUC (Animal Welfare Assurance A3472-01) or Princeton University IACUC (Animal Welfare Assurance D16-00273). All work was performed with the design in mind for minimal pain and animal welfare at the forefront. In the event of severe disease or distress, mice were promptly euthanized. C57BL/6J mice and mice *Irf1*$^{-/-}$ mice were obtained from Jackson Laboratory (Strain: #000664 and #002762, respectively). Additionally, *Irf1*$^{\text{flox/flox}}$ mice (V. Tarakanova, Medical College of Wisconsin) mice were crossed with LysM Cre mice (Strain: #004781) to generate *Irf1*$^{\Delta LysM}$ and *Irf1*$^{fl/fl}$ littermate controls for experimental use. Whole body non-functional *Clock*$^{\Delta 19}$ mice (missing exon 19 in CLOCK; dominant negative mutation) were obtained from J. Takahashi (UT Southwestern Medical Center). Mice were housed in 12-hour light/ 12-hour dark cycle within specific pathogen-free (SPF) barrier facilities prior to experiments. 8–12-week-old mice were used in all experiments. Any modulations of lighting conditions used isolation cabinets with green LED lights programmed by ClockLab v3.604 and Chamber Control Software v4.114 (Actimetrics Inc., Wilmette, IL).

### Infections and plaque assays

Mice were infected by peroral infection ($10^9$ plaque forming units of CVB3-H3 in 25 ul pipetted into the mouth) or intraperitoneal injection ($10^5$ plaque forming units of CVB3-H3 in 100 ul). Additionally, due to a previously published a sex bias in murine CVB3 infection, only male mice were used for infection experiments *(26)*. All infections took place at Zeitgeber times (ZT) 0, 4, 8, 12, 16, or 20. 24 hours after infection, ileum, cecum, and stool samples from each mouse were harvested for further analysis. At 24h post-infection, tissues or stool were weighed and homogenized using a bead beater homogenizer, followed by three freeze-thaw cycles to release virus prior to plaque assay using HeLa cells *(39)*. Briefly, dilutions of virus were used to infect HeLa cell monolayers which were then covered with 1% agarose overlays. At two days post infection, cells were fixed and stained with a crystal violet solution and plaques were counted.

### qRT-PCR Gene Expression Experiments

Murine samples were harvested and placed in 1mL TRIzol™ Reagent. To extract RNA, samples were homogenized and extracted using the Qiagen RNeasy® Plus Universal Mini Kit. cDNA synthesis began by adding 10ng of sample RNA to water (for 14 µL total), 1 µL Random Hexamers, 1 µL dNTP, and heating to 65°C for 5 minutes. Samples were then immediately

bioRxiv preprint doi: https://doi.org/10.64898/2026.05.15.725500; this version posted May 16, 2026. The copyright holder for this preprint (which was not certified by peer review) is the author/funder, who has granted bioRxiv a license to display the preprint in perpetuity. It is made available under a CC-BY 4.0 International license.

chilled on ice for 2 minutes. Next, to each tube 5 µL 5x first-stand buffer, 2 µL 0.1M DTT, and 1 µL RNAaseOUT were added to each sample and incubated at 37°C for 2 minutes. Following this, 1 µL of M-MLV RT was added to each sample and the tubes were incubated using the MMV1 method. Following cDNA synthesis, samples were examined with quantitative PCR. For each reaction, 1 µL of cDNA was added to 10 µL Platinum™ SYBR™ Green qPCR SuperMix-UDG, 1 µL BSA (20x 1mg/mL), 5.9 µL nuclease free water, 0.1µL ROX Reference Dye, 1 µL of 0.5 µM forward primer, and 1 µL of 0.5 µM reverse primer. Each 20 µL reaction was added into a 384 well plate and incubated using the QS7Pro-384-Well-PCR-Melt-Std method on the Quant Studio™ 7 Pro qPCR machine. Cq scores were analyzed using Design & Analysis (v1 4.3 download) as well as Microsoft Excel (Version 2510, build 19328.20266).

**Cloning**
gBlock sequences were obtained from IDT. Sequences were cloned into a luciferase reporter vector using the New England Biolabs Gibson Assembly® Master Mix. Following colony qPCR and plasmid purification via the Qiagen QIAprep® Spin Miniprep Kit, plasmids from candidate colonies were sent for Sanger sequencing to GENEWIZ from Azenca. Candidate plasmids were aligned to desired sequence and matching sequences were preserved as glycerol stocks.

**Luciferase Reporter Assays**
Human Embryonic Kidney (HEK) cells were plated on a 24-well dish and grown to 70% confluency. Cells were transfected with 500 ng of each plasmid using the FuGENE® HD Transfection Reagent. A Human Luciferase/Renilla Luciferase plasmid and untransfected cells were used as controls. 48 hours after the initial transfection, Firefly and Renilla Luciferase luminescence was measured in each well using the Dual-Glo® Luciferase Assay System, a Tecan Infinite® 200 PRO plate reader, and the i-control™ software.

***In vitro* chromatin immunoprecipitation (ChIP)**
Human Embryonic Kidney (HEK) cells were plated on a 6-well dish and grown to 70% confluency. Cells were transfected with 1,000ng of each plasmid using the FuGENE® HD Transfection Reagent. 48 hours after the initial transfection, cells were fixed in 1% paraformaldehyde at 37°C for 10 minutes, quenched in 1M glycine at room temperature for 10 minutes, and then washed in PBS+ 1x protease inhibitor + 1x phosphatase inhibitor. Next, using an Eppendorf rotator, cells were lysed in Lysis Buffer 1 (4°C for 10 minutes, then centrifuged at 4°C, 1,350g for 5 minutes) and Lysis Buffer 2 (room temperature for 10 minutes, centrifuged at 4°C, 1,350g for 5 minutes). Following this, cells were resuspended in Lysis Buffer 3 (ThermoFisher Scientific, #00-4333-57), sonicated (Diagenode Bioruptor Plus) for 9 cycles at 20 seconds on and 30 seconds off, and centrifuged at 4°C, 1,350g for 5 minutes. To solubilize the nuclear membrane, 1/10 volume of 10% Triton X-100 was then added to samples. To crack the nuclei, samples were centrifuged at 4°C, 20,000g for 10 minutes and the supernatant (nuclear content) was retained. DNA concentration was quantified using the Qubit 1X dsDNA kit. The chromatin immunoprecipitation reaction consisted of 1 mg of shared chromatin and 2.5 mg of either BMAL1 (Cell Signaling Technology, D2L7G Rabbit Monoclonal Antibody #14020), or IgG (Cell Signaling Technology, Normal Rabbit IgG #2729). The initial reaction was incubated at room temperature for 1 hour on the Eppendorf rotator. Next, Pierce™ Protein A/G Magnetic Beads were cleared in TBS-T buffer and then added to each chromatin immunoprecipitation reaction (25 µL) for 1 hour. The beads were then washed in RIPA buffer (ThermoFisher Scientific, #89901), washed in 1 mL TE 50 mM NaCl, and then eluted in Pierce™ Gentle Ag/Ab Elution Buffer, pH 6.6 at 65°C for 30 minutes. To reverse crosslinks, samples were incubated in

bioRxiv preprint doi: https://doi.org/10.64898/2026.05.15.725500; this version posted May 16, 2026. The copyright holder for this preprint (which was not certified by peer review) is the author/funder, who has granted bioRxiv a license to display the preprint in perpetuity. It is made available under a CC-BY 4.0 International license.

RNase A at 37°C for 30 minutes and then incubated in Proteinase K at 57°C overnight. The following day, the DNA was purified using the Qiagen QIAquick® PCR Purification Kit and analyzed by qPCR primer probes (for CLOCK/BMAL1 E-boxes or IRF Response Element). Cq scores were analyzed using Design & Analysis (v1 4.3 download) as well as Microsoft Excel (Version 2510, build 19328.20266), and analyzed using the fold-change method.

### *In vivo* chromatin immunoprecipitation (ChIP)

Murine intestinal samples were harvested, cut into 0.5cmx0.5cm sections, and washed in cold PBS. Next, samples were placed in 15mL conical tubes, fixed in 1% paraformaldehyde at 37°C for 10 minutes, quenched in 1M glycine, pH 3.2, at room temperature for 10 minutes, and then washed in PBS+ 1x protease inhibitor + 1x phosphatase inhibitor. Next, using an Eppendorf rotator, samples were lysed in Lysis Buffer 1 [consisting of: 50 mM HEPES-KOH (pH 7.5; HEPES: ThermoFisher Scientific, Gibco™, 15630080; KOH: ThermoFisher Scientific, 437135000), 140 mM NaCl (ThermoFisher Scientific, AAJ2161836), 1 mM EDTA (ThermoFisher Scientific, Invitrogen™, 15575020), 10% glycerol (ThermoFisher Scientific, A16205.0F), 0.5% NP-40 (ThermoFisher Scientific, 85124), and 0.25% Triton X-100 (ThermoFisher Scientific, 85111), prepared in $ddH_2O$.] (4°C for 10 minutes, then centrifuged at 4°C, 1,350g for 5 minutes) and Lysis Buffer 2 [consisting of 10 mM Tris-HCl (pH 8.0; ThermoFisher Scientific, Invitrogen™, 12090015), 200 mM NaCl (ThermoFisher Scientific, AAJ2161836), 1 mM EDTA (ThermoFisher Scientific, Invitrogen™, 15575020), and 0.5 mM EGTA (Millipore Sigma, 324626-25GM), supplemented with protease and phosphatase inhibitors (Halt™ Protease Inhibitor Cocktail, 100X, 78430; Halt™ Phosphatase Inhibitor Cocktail, 78420; ThermoFisher Scientific), prepared in $ddH_2O$] (room temperature for 10 minutes, centrifuged at 4°C, 1,350g for 5 minutes). Following this, cells were resuspended in Lysis Buffer 3, sonicated (Diagenode Bioruptor Plus) for 12 cycles at 30 seconds on and 30 seconds off, and centrifuged at 4°C, 1,350g for 5 minutes. To solubilize the nuclear membrane, 1/10 volume of 10% Triton X-100 was then added to samples. To crack the nuclei, samples were centrifuged at 4°C, 20,000g and the supernatant (nuclear content) was retained. DNA concentration was quantified using the Qubit 1X dsDNA kit. The chromatin immunoprecipitation reaction consisted of 1 mg of shared chromatin and 2.5 mg of either BMAL1 (Cell Signaling Technology, D2L7G Rabbit Monoclonal Antibody #14020), or IgG (Cell Signaling Technology, Normal Rabbit IgG #2729). The initial reaction was incubated at room temperature for 1 hour on the Eppendorf rotator. Next, Pierce™ Protein A/G Magnetic Beads were cleared in TBS-T buffer and then added to each chromatin immunoprecipitation reaction (25 µL) for 1 hour. The beads were then washed in RIPA buffer, washed in 1 mL TE 50 mM NaCl, and then eluted in Pierce™ Gentle Ag/Ab Elution Buffer, pH 6.6 at 65°C for 30 minutes. To reverse crosslinks, samples were incubated in RNase A at 37°C for 30 minutes and then incubated in Proteinase K at 57°C overnight. The following day, the DNA was purified using the Qiagen QIAquick® PCR Purification Kit and analyzed by qPCR primer probes (for *Irf1* E-boxes and Exon 1). Cq scores were analyzed using Design & Analysis (v1 4.3 download) as well as Microsoft Excel (Version 2510, build 19328.20266), and analyzed using both the fold-change and percent input methods.

### Western Blot

Mouse ileal tissue was homogenized in T-PER Tissue Protein Extraction Reagent+ protease + phosphatase inhibitor tablets. For each sample, 40 mg of protein was loaded into a 4-20% Mini-PROTEAN® TGX™ protein gel and transferred to a PVDF membrane. Next, membranes were blocked in 5% dry nonfat milk in PBS-T for 1 hour. Membranes were incubated in the following

bioRxiv preprint doi: https://doi.org/10.64898/2026.05.15.725500; this version posted May 16, 2026. The copyright holder for this preprint (which was not certified by peer review) is the author/funder, who has granted bioRxiv a license to display the preprint in perpetuity. It is made available under a CC-BY 4.0 International license.

primary antibodies (Cell Signaling Technology, D5E4 (IRF1) Monoclonal Antibody #8478; Cell Signaling Technology, OAS2 Antibody #54155) at 4°C overnight in 1:1,000 dilutions. The following day, membranes were washed in PBS-T. Membranes were then incubated in HRP-conjugated secondary antibody (Cell Signaling Technology, Anti-rabbit IgG, HRP-linked Antibody #7074) at room temperature for 2 hours in 1:10,000 dilutions. A Bio-Rad ChemiDoc™ Imaging System was used to visualize membranes.

**Food Consumption Measurements**

Food intake and feeding times were measured using a metabolic cage (Labmaster, TSE Systems GmbH, Germany). Wildtype and *Irf1*$^{-/-}$ mice were individually housed in a light and temperature (22.5–23.5°C) controlled environment. Following a five-day acclimation in the home cage, mice were analyzed in the metabolic chambers for six days. All mice were provided with food (LabDiet, 5K52) and water ad libitum; food and water intake were continuously recorded using lid-mounted sensors.

**RNA-seq**

Murine ileal samples were harvested at ZT0 and placed in 1mL TRIzol™ Reagent. To extract RNA, samples were homogenized and extracted using the Qiagen RNeasy® Plus Universal Mini Kit. Sample quality was analyzed using the Qubit™ RNA High Sensitivity (HS) and the Agilent 2100 Bioanalyzer. Following quality control, an RNA-seq Directional Library was prepared on the Apollo 324 robot. Next, bulk RNA-sequencing was performed using the NovaSeq SP 100nt Flowcell v 1.5 protocol. Following a FastQC file quality check, sample reads were aligned to the UCSC reference mouse genome (mm39) in R (version 4.5.2). Differential Gene Expression Counts were generated using the DESeq2 package in R. Reads with low mapping quality (less than 10 recounts) were filtered out.

**Lamina Propria and CSF1R Depletion Experiment**

For the macrophage depletion experiment, wildtype mice were injected at ZT4 with 250 µL of antibodies to the CSF1R receptor (BioXCell, BE0213) or IgG as a control (BioXCell, BE0089). 24 hours after the initial injection, all mice were injected again with the same antibody as the previous day. 24 hours after the second injection, animals were sacrificed and terminal ileum samples were collected for a lamina propria isolation.

Murine terminal ileum was collected and placed in PBS+2%FBS; Peyer's patches were removed from samples. IECs were isolated using EDTA-DTT Buffer at 37°C for 15 minutes. IECs were placed in TRIzol™ Reagent for subsequent gene expression analysis. To isolate the lamina propria, samples (not containing IECs) were incubated in Digest Solution at 37°C, 250 RPM for 1 hour. Using the 40% and 80% Percoll solutions, a Percoll gradient was established to extract cells in the lamina propria. Lamina propria cells were placed in TRIzol™ Reagent for subsequent gene expression analysis.

**Promoter Analysis**

Promoter analysis for E-boxes and IREs was performed using the Genome Reference Consortium Mouse Build 39 (GRCm39/mm39) reference genome in the University of California, Santa Cruz browser.

**Statistical Analysis**

bioRxiv preprint doi: https://doi.org/10.64898/2026.05.15.725500; this version posted May 16, 2026. The copyright holder for this preprint (which was not certified by peer review) is the author/funder, who has granted bioRxiv a license to display the preprint in perpetuity. It is made available under a CC-BY 4.0 International license.

Plaque assays for two conditions were examined for normality to determine which unpaired T-test (Mann-Whitney) was used. Plaque assays using more than two conditions used one-way ANOVA with multiple comparisons with Tukey corrections. Furthermore, CircaCompare was used to detect rhythmicity, determine peak and trough of the data, and establish a best-fit cosinor line values. Rhythms with a p-value less than 0.0001 were defined as significantly rhythmic *(40)*.

bioRxiv preprint doi: https://doi.org/10.64898/2026.05.15.725500; this version posted May 16, 2026. The copyright holder for this preprint (which was not certified by peer review) is the author/funder, who has granted bioRxiv a license to display the preprint in perpetuity. It is made available under a CC-BY 4.0 International license.

## Figure Legends

**Fig. S1. Time of day determines host susceptibility to enteric viral infection in the cecum.**

**(A)** CircaCompare analysis of ileum, cecum, and stool following peroral infection with $10^9$ PFU CVB3 at six timepoints across the day-night cycle. Data was double plotted for visualization and rhythmicity was assessed via CircaCompare in R ($P < 0.0001$).
**(B)** Mice were perorally infected with $10^9$ PFU CVB3 at six timepoints across the day-night cycle and viral load was quantified in the cecum at 24h post-infection. Black dashed line shows rhythmicity analysis of peak and trough within the data set. Gray dotted line indicates detection limit.
**(C)** Quantification of viral load in cecum 24h post-infection in wildtype C57BL/6 mice from The Jackson Laboratory versus Taconic Biosciences.
**(D)** Quantification of viral load in the cecum 24h post-infection following intraperitoneal (IP) injection in wildtype mice.
**(E)** Quantification of viral load in the cecum 24h post infection in *Clock*$^{\Delta19/\Delta19}$ mice.

Best fit curves for rhythmicity are plotted. ****$P < 0.0001$ as determined by CircaCompare.

**Fig. S2. Expression of certain host genes is rhythmic in uninfected mice.**

**(A)** qRT-PCR quantification of *Irf3, Irf5, Irf7,* and *Irf8* at six timepoints across the day-night cycle.
**(B)** CircaCompare analysis of gene expression for *Bmal1* and antiviral genes (*Stat1*, *Stat2*, and *Irf1-Irf9).* Data was double plotted for visualization and rhythmicity was assessed via CircaCompare in R.
**(C)** CircaCompare analysis of *Irf1* gene expression in *Clock* $^{\Delta19/\Delta19}$ mice. Data were double plotted for visualization and rhythmicity was assessed via CircaCompare.

Best fit curve for rhythmic genes are plotted. ****$P < 0.0001$ as determined by CircaCompare.

**Fig. S3. Confirmation of IRF1 loss and circadian clock preservation in *Irf1*$^{-/-}$ mice.**

**(A)** qRT-PCR quantification of the *Irf1* DNA-Binding Domain at ZT0 in wildtype and *Irf1*$^{-/-}$ mice.
**(B)** Western blot quantification of IRF1 at ZT0 in wildtype and *Irf1*$^{-/-}$ mice.
**(C)** Food consumed across the day-night cycle in wildtype and *Irf1*$^{-/-}$ mice.
**(D)** CircaCompare examination of *Per2* across the day-night cycle in wildtype ($P < 0.0001$) and *Irf1*$^{-/-}$ ($P < 0.0001$) mice.
**(E)** Gene ontology for Top 25 differentially enriched pathways in wildtype vs. *Irf1*$^{-/-}$ mice.
**(F)** CircaCompare examination of *Oas2* across the day-night cycle in wildtype ($P < 0.0001$) and *Irf1*$^{-/-}$ ($P < 0.0001$) mice.

Means ± SEM are plotted; **$P < 0.01$; as determined by Student's t-test .
For rhythmic data, best fit curves for rhythmicity are plotted. ****$P < 0.0001$ as determined by CircaCompare.

bioRxiv preprint doi: https://doi.org/10.64898/2026.05.15.725500; this version posted May 16, 2026. The copyright holder for this preprint (which was not certified by peer review) is the author/funder, who has granted bioRxiv a license to display the preprint in perpetuity. It is made available under a CC-BY 4.0 International license.

# Supplemental Figure 1

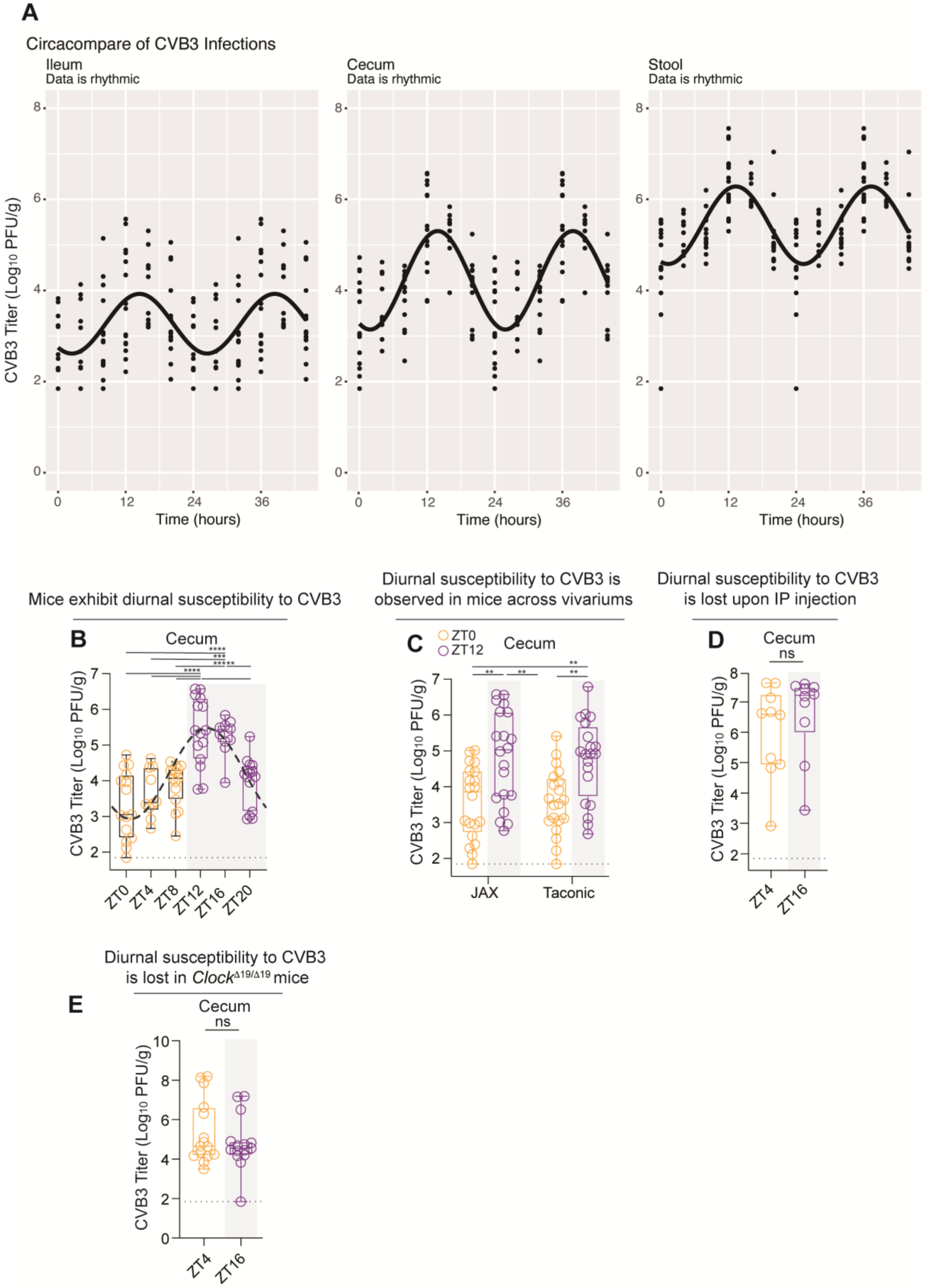

bioRxiv preprint doi: https://doi.org/10.64898/2026.05.15.725500; this version posted May 16, 2026. The copyright holder for this preprint (which was not certified by peer review) is the author/funder, who has granted bioRxiv a license to display the preprint in perpetuity. It is made available under a CC-BY 4.0 International license.

## Supplemental Figure 2.

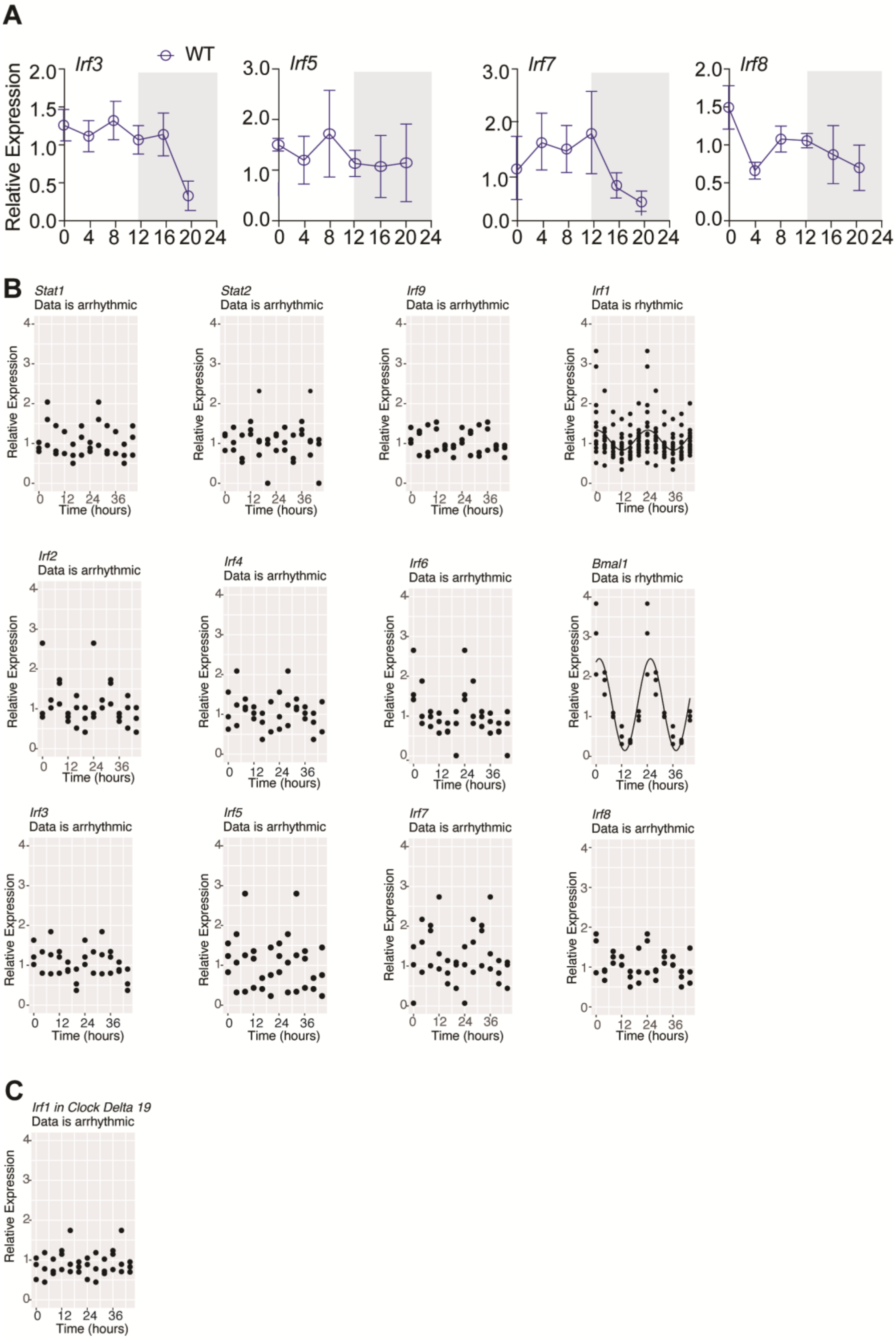

bioRxiv preprint doi: https://doi.org/10.64898/2026.05.15.725500; this version posted May 16, 2026. The copyright holder for this preprint (which was not certified by peer review) is the author/funder, who has granted bioRxiv a license to display the preprint in perpetuity. It is made available under a CC-BY 4.0 International license.

## Supplemental Figure 3.

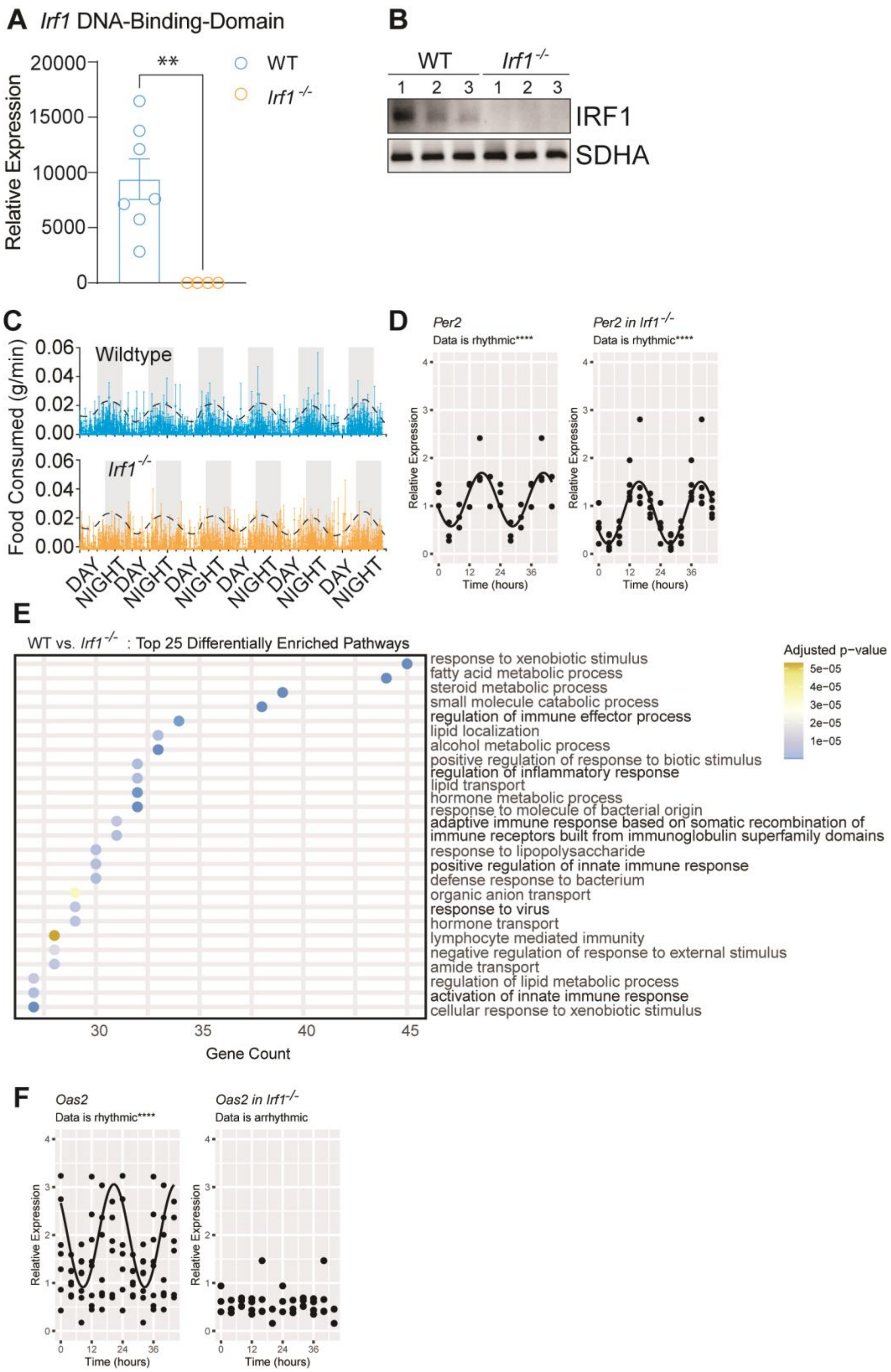

bioRxiv preprint doi: https://doi.org/10.64898/2026.05.15.725500; this version posted May 16, 2026. The copyright holder for this preprint (which was not certified by peer review) is the author/funder, who has granted bioRxiv a license to display the preprint in perpetuity. It is made available under a CC-BY 4.0 International license.

**Table S1: Mice**

| **Item** | **Vendor** | **Catalog Number** |
|---|---|---|
| C57BL/6J wildtype Mice | The Jackson Laboratory | 00664 |
| B6.129S2-Irf1tm1Mak/J | The Jackson Laboratory | 002762 |
| B6.129-Bmal1tm1Bra/J | The Jackson Laboratory | 009100 |
| C57BL/6J Irf1$^{fl/fl}$ | Dr. Vera Tarakanova | NA |
| C.B6-Clockm1Jt/J | The Jackson Laboratory | 016175 |
| B6.129P2-Lyz2tm1(cre)Ifo/J | The Jackson Laboratory | 004781 |
| C57BL/6NTac | Taconic Biosciences | B6-M |

**Table S2: Cell Culture and Virus Infection Reagents**

| **Item** | **Vendor** | **Catalog Number** |
|---|---|---|
| HeLa Cells | Dr. Karla Kirkegaard | NA |
| CVB3 H3 Clone | Dr. Marco Vignuzzi | NA |
| Dulbecco’s Modified Eagle’s Medium (DMEM), High Glucose | ThermoFisher Scientific | 11965118 |
| Newborn Calf Serum (Heat Inactivated) | ThermoFisher Scientific | NC1928575 |
| Lipofectamine 2000 Transfection Reagent 1.5mL | ThermoFisher Scientific | 11668019 |
| Phosphate-buffered saline, no calcium or magnesium (PBS) | ThermoFisher Scientific, Gibco™ | 14190144 |

**Table S3: Tissue Processing and Plaque Assay Reagents**

| **Item** | **Vendor** | **Catalog Number** |
|---|---|---|
| Fisherbrand™ Bead Mill 4 Mini Homogenizer | Fisherbrand™ | 15-340-164 |
| Omni Bulk Beads, Stainless Steel 2.4mm | Millipore Sigma | MNI-19-640 |
| Dulbecco's phosphate-buffered saline with calcium and magnesium (PBS+) | ThermoFisher Scientific, Gibco™ | 14040133 |

bioRxiv preprint doi: https://doi.org/10.64898/2026.05.15.725500; this version posted May 16, 2026. The copyright holder for this preprint (which was not certified by peer review) is the author/funder, who has granted bioRxiv a license to display the preprint in perpetuity. It is made available under a CC-BY 4.0 International license.

| | | |
|---|---|---|
| Phosphate-buffered saline, no calcium or magnesium (PBS) | ThermoFisher Scientific, Gibco™ | 14190144 |
| Dulbecco's Modified Eagle's Medium (DMEM), High Glucose | ThermoFisher Scientific | 11965118 |
| Sodium bicarbonate powder 500g | Millipore Sigma | S8875-500G |
| SeaKem LE Agarose 500g | Lonza | BMA50000 |
| Crystal Violet | Millipore Sigma | C0775-25G |

**Table S4: qRT-PCR Gene Expression Experiments Reagents**

| **Item** | **Vendor** | **Catalog Number** |
|---|---|---|
| TRIzol™ | ThermoFisher Scientific, Invitrogen™ | 15596026 |
| Qiagen RNeasy® Plus Universal Mini Kit | Qiagen | 73404 |
| Random Hexamers 50 uM | ThermoFisher Scientific, Invitrogen™ | N8080127 |
| 10 mM dNTP Mix | ThermoFisher Scientific, Invitrogen™ | 18427013 |
| 5x First-Stand Buffer | ThermoFisher Scientific, Invitrogen™ | 18057018 |
| DTT | ThermoFisher Scientific, Invitrogen™ | 18057018 |
| M-MLV RT | ThermoFisher Scientific, Invitrogen™ | 18057018 |
| RNaseOUT™ Recombinant Ribonuclease Inhibitor | ThermoFisher Scientific, Invitrogen™ | 10777019 |
| Platinum™ SYBR™ Green qPCR SuperMix-UDG (includes ROX and BSA) | ThermoFisher Scientific, Invitrogen™ | 11733046 |
| ROX Reference Dye | ThermoFisher Scientific, Invitrogen™ | 12223012 |
| HyClone™ HyPure Water, Molecular Biology Grade | Cytiva | SH30538.FS |

bioRxiv preprint doi: https://doi.org/10.64898/2026.05.15.725500; this version posted May 16, 2026. The copyright holder for this preprint (which was not certified by peer review) is the author/funder, who has granted bioRxiv a license to display the preprint in perpetuity. It is made available under a CC-BY 4.0 International license.

| | | |
|---|---|---|
| Applied Biosystems™ QuantStudio™ 7 Pro Real-Time PCR System, 384-well | Fisher Scientific | A43171 |

**Table S5: Cloning Reagents**

| **Item** | **Vendor** | **Catalog Number** |
|---|---|---|
| Gibson Assembly® Master Mix | New England Biolabs | E2611S |
| Qiagen QIAprep® Spin Miniprep Kit | Qiagen | 27106 |
| NEB® 10-beta Electrocompetent E. coli | New England Biolabs | C3020K |
| Glycerol, 99+% | ThermoFisher Scientific | A16205.0F |

**Table S6: Luciferase Reporter Assays Reagents**

| **Item** | **Vendor** | **Catalog Number** |
|---|---|---|
| HEK 293T Cells | ATCC | CRL-3216 ™ |
| Dulbecco’s Modified Eagle’s Medium (DMEM), High Glucose | ThermoFisher Scientific | 11965118 |
| Fetal Bovine Serum (Heat Inactivated) | Millipore Sigma (Sigma Aldrich) | F4135-500ML |
| MEM Non-Essential Amino Acids Solution (100X) | ThermoFisher Scientific, Gibco™ | 11140050 |
| GlutaMAX™ Supplement | ThermoFisher Scientific, Gibco™ | 35050061 |
| Sodium Pyruvate (100 mM) | ThermoFisher Scientific, Gibco™ | 11360070 |
| Penicillin-Streptomycin (10,000 U/mL) | ThermoFisher Scientific, Gibco™ | 15140122 |
| Phosphate-buffered saline, no calcium or magnesium (PBS) | ThermoFisher Scientific, Gibco™ | 14190144 |
| Costar® Costar 24-Well Clear TC Treated Plates | Corning® | 3524 |
| FuGENE® HD Transfection Reagent. | Promega | E2311 |

bioRxiv preprint doi: https://doi.org/10.64898/2026.05.15.725500; this version posted May 16, 2026. The copyright holder for this preprint (which was not certified by peer review) is the author/funder, who has granted bioRxiv a license to display the preprint in perpetuity. It is made available under a CC-BY 4.0 International license.

| | | |
|---|---|---|
| Opti-MEM™ I Reduced Serum Medium | ThermoFisher Scientific, Gibco™ | 31985088 |
| 24-Well Black Visiplate TC, Case of 14 | Revvity | 1450-605 |
| Thermo Scientific MaxQ 416 High Performance Orbital Shaker | ThermoFisher Scientific | SHKE416HP |
| Dual-Glo® Luciferase Assay System | Promega | E2940 |
| Tecan Infinite® 200 PRO plate reader | Tecan | M Plex |
| pLX304_zeo_mmIrf1 Plasmid | Addgene | 160098 |
| pBMPC3 Bmal1 Plasmid | Addgene | 31367 |
| pCKFB1 Clock Plasmid | Addgene | 31280 |

**Table S7: Lysis Buffer 1 (40 mL)**

| Volume | Reagent | | Final Concentration |
|---|---|---|---|
| 2 mL | 1M Hepes-KOH, pH 7.5 | → | 50 mM |
| 1,120 µL | 5M NaCl | → | 140 mM |
| 80 µL | 0.5M EDTA | → | 1 mM |
| 8 mL | 50% Glycerol | → | 10% |
| 2 mL | 10% NP-40 | → | .5% |
| 1 mL | 10% Triton X-100 | → | .25% |
| 25.8 mL | $ddH_2O$ | | |

**Table S8: Lysis Buffer 2 (40 mL)**

| Volume | Reagent | | Final Concentration |
|---|---|---|---|
| 400 µL | 1M Tris-HCl, pH 8.0 | → | 10 mM |
| 1,600 µL | 5M NaCl | → | 200 mM |
| 80 µL | 0.5M EDTA | → | 1 mM |
| 40 µL | 0.5M EGTA | → | 0.5 mL=M |

bioRxiv preprint doi: https://doi.org/10.64898/2026.05.15.725500; this version posted May 16, 2026. The copyright holder for this preprint (which was not certified by peer review) is the author/funder, who has granted bioRxiv a license to display the preprint in perpetuity. It is made available under a CC-BY 4.0 International license.

| | | | |
|---|---|---|---|
| 37.8 µL | $ddH_2O$ | | |

**Table S9: Chromatin Immunoprecipitation (ChIP) Reagents**

| Item | Vendor | Catalog Number |
|---|---|---|
| Paraformaldehyde Solution, 4% in PBS | ThermoFisher Scientific | J19943.K2 |
| Glycine, 99% | ThermoFisher Scientific | A13816.36 |
| Halt™ Protease Inhibitor Cocktail (100X) | ThermoFisher Scientific | 78430 |
| Halt™ Phosphatase Inhibitor Single-Use Cocktail | ThermoFisher Scientific | 78420 |
| Tube rotator revolver | ThermoFisher Scientific, Fisherbrand™ | 05-450-127 |
| 1M Hepes | ThermoFisher Scientific, Gibco™ | 15630080 |
| Potassium hydroxide pellets | ThermoFisher Scientific | 437135000 |
| Sodium Chloride, ACS, ≥99%, Ultrapure | ThermoFisher Scientific | AAJ2161836 |
| UltraPure™ 0.5M EDTA, pH 8.0 | ThermoFisher Scientific, Invitrogen™ | 15575020 |
| Glycerol, 99+% | ThermoFisher Scientific | A16205.0F |
| NP-40 Surfact-Amps™ Detergent Solution | ThermoFisher Scientific | 85124 |
| Triton™ X-100 Surfact-Amps™ Detergent Solution | ThermoFisher Scientific | 85111 |
| Tris (1 M), pH 8.0, RNase-free | ThermoFisher Scientific, Invitrogen™ | AM9855G |
| EGTA, Molecular Biology Grade (25g) | Millipore Sigma | 324626-25GM |
| Pierce™ IP Lysis Buffer | ThermoFisher Scientific | 87787 |
| Diagenode Bioruptor Plus | Diagenode | B01020001 |
| Centrifuge 5420 - Centrifuge for Eppendorf tubes | Eppendorf | 5420000245 |
| Qubit 1X dsDNA | ThermoFisher Scientific | Q33231 |

bioRxiv preprint doi: https://doi.org/10.64898/2026.05.15.725500; this version posted May 16, 2026. The copyright holder for this preprint (which was not certified by peer review) is the author/funder, who has granted bioRxiv a license to display the preprint in perpetuity. It is made available under a CC-BY 4.0 International license.

| | | |
|---|---|---|
| BMAL1 (D2L7G) Rabbit Monoclonal Antibody | Cell Signaling Technology | 14020 |
| IRF-1 (D5E4) Rabbit Monoclonal Antibody | Cell Signaling Technology | 8478 |
| Normal Rabbit IgG Antibody | Cell Signaling Technology | 2729 |
| Pierce™ Protein A/G Magnetic Beads | ThermoFisher Scientific | 88802 |
| TRIS-buffered saline (TBS, 10X) pH 7.4 | ThermoFisher Scientific | J60764.K2 |
| Tween™ 20 Surfact-Amps™ Detergent Solution | ThermoFisher Scientific | PI85114 |
| RIPA Lysis and Extraction Buffer | ThermoFisher Scientific | 89901 |
| TE Buffer | ThermoFisher Scientific, Invitrogen™ | 12090015 |
| Pierce™ Gentle Ag/Ab Elution Buffer, pH 6.6 | ThermoFisher Scientific | 21027 |
| Monarch® RNase A | New England Biolabs | T3018L |
| Proteinase K, Molecular Biology Grade | New England Biolabs | P8107S |
| Qiagen QIAquick® PCR Purification Kit | Qiagen | 28106 |

**Table S10: Western Blot Reagents**

| **Item** | **Vendor** | **Catalog Number** |
|---|---|---|
| T-PER™ Tissue Protein Extraction Reagent | ThermoFisher Scientific | 78510 |
| Pierce™ Protease Inhibitor Tablets, EDTA-free | ThermoFisher Scientific | A32965 |
| Thermo Scientific™ Pierce™ Phosphatase Inhibitor Mini Tablets | ThermoFisher Scientific | A32957 |
| 10x Tris/Glycine/SDS Buffer 1L | Bio-Rad | 1610732 |
| Novex™ Tris-Glycine SDS Sample Buffer (2X) | ThermoFisher Scientific, Invitrogen™ | LC2676 |
| Mini-PROTEAN® TGX™ Protein Gel | Bio-Rad | 4561094 |
| PVDF Transfer Membranes, 0.45 µm, 1 Roll | ThermoFisher Scientific | PI88518 |

bioRxiv preprint doi: https://doi.org/10.64898/2026.05.15.725500; this version posted May 16, 2026. The copyright holder for this preprint (which was not certified by peer review) is the author/funder, who has granted bioRxiv a license to display the preprint in perpetuity. It is made available under a CC-BY 4.0 International license.

| | | |
|---|---|---|
| Blotting Grade Blocker Non-Fat Dry Milk | Bio-Rad | 1706404XTU |
| Phosphate-buffered saline, no calcium or magnesium (PBS) | ThermoFisher Scientific, Gibco™ | 14190144 |
| Tween™ 20 Surfact-Amps™ Detergent Solution | ThermoFisher Scientific | PI85114 |
| BMAL1 (D2L7G) Rabbit Monoclonal Antibody | Cell Signaling Technology | 14020 |
| IRF-1 (D5E4) Rabbit Monoclonal Antibody | Cell Signaling Technology | 8478 |
| β-Actin (13E5) Rabbit mAb | Cell Signaling Technology | 4970 |
| OAS2 Antibody | Cell Signaling Technology | 54155 |
| SDHA Monoclonal Antibody | ThermoFisher Scientific, Invitrogen™ | 459200 |
| Anti-rabbit IgG, HRP-linked Antibody | Cell Signaling Technology | 7074S |

**Table S11: RNA-seq Reagents**

| **Item** | **Vendor** | **Catalog Number** |
|---|---|---|
| TRIzol™ | ThermoFisher Scientific, Invitrogen™ | 15596026 |
| Qiagen RNeasy® Plus Universal Mini Kit | Qiagen | 73404 |
| Qubit™ RNA High Sensitivity (HS) | ThermoFisher Scientific, Invitrogen™ | Q32852 |
| Bioanalyzer High Sensitivity RNA Analysis | Agilent | 5067-1513 |
| Illumina Stranded Total RNA Prep | Illumina | M-GL-02148 v1.0 |
| Illumina RNA Prep with Enrichment | Illumina | M-GL-02145 v1.0 |

bioRxiv preprint doi: https://doi.org/10.64898/2026.05.15.725500; this version posted May 16, 2026. The copyright holder for this preprint (which was not certified by peer review) is the author/funder, who has granted bioRxiv a license to display the preprint in perpetuity. It is made available under a CC-BY 4.0 International license.

**Table S12: Immunofluorescence Examination Reagents**

| **Item** | **Vendor** | **Catalog Number** |
|---|---|---|
| Bouin's Fixative | ThermoFisher Scientific | 88038 |
| ParaFilm® M Lab Film - 5 Mil, 4" x 125' | Uline | S-25929 |
| Xylene | ThermoFisher Scientific | X3P1GAL |
| Ethanol, Pure, 200 Proof (100%), USP, KOPTEC | Avantor Sciences | 89125-172 |
| Citric acid monohydrate | Millipore Sigma | C1909 |
| Distilled Water | ThermoFisher Scientific | 15230147 |
| Phosphate-buffered saline, no calcium or magnesium (PBS) | ThermoFisher Scientific, Gibco™ | 14190144 |
| BSA | Millipore Sigma | A9418 |
| TRIS-buffered saline (TBS, 10X) pH 7.4 | ThermoFisher Scientific | J60764.K2 |
| Triton™ X-100 Surfact-Amps™ Detergent Solution | ThermoFisher Scientific | 85111 |
| AlexaFluor 488 Donkey anti-rabbit IgG | Abcam | Ab150073 |
| Texas Red Goat Anti-Armenian Hamster IgG | Abcam | Ab5743 |
| DAPI and Hoechst Nucleic Acid Stains Share | ThermoFisher Scientific, Invitrogen™ | D1306 |
| Fluoromount-G ® | ThermoFisher Scientific, Invitrogen™ | 00-4958-02 |
| Prolong Diamond Antifade | ThermoFisher Scientific | P36965 |
| Keyence Fluorescence Microscope BZ-X800 | Keyence | BZ-X800 |

**Table S13: CSF1R Depletion Experiment Reagents**

bioRxiv preprint doi: https://doi.org/10.64898/2026.05.15.725500; this version posted May 16, 2026. The copyright holder for this preprint (which was not certified by peer review) is the author/funder, who has granted bioRxiv a license to display the preprint in perpetuity. It is made available under a CC-BY 4.0 International license.

| Item | Vendor | Catalog Number |
|---|---|---|
| InVivoMAb anti-mouse CSF1R (CD115) | BioXCell | BE0213 |
| InVivoMAb rat IgG2a isotype control, anti-trinitrophenol | BioXCell | BE0089 |

**Table S14: Lamina Propria Isolation Buffers**

| Buffer | Reagents |
|---|---|
| EDTA-DTT Buffer | 14.25 mL HBSS<br>300 µL of 0.5M EDTA<br>150 µL of 1M DTT<br>300 µL FBS |
| Digest Solution | 4.82 mL RPMI<br>31.25 µL Liberase<br>250 µL DNase I<br>100 µL FBS |
| 80% Percoll | 2 mL Percoll<br>250 µL HBSS<br>250 µL RNase Free $H_2O$ |

**Table S15: Lamina Propria Isolation Reagents**

| Item | Vendor | Catalog Number |
|---|---|---|
| HBSS, no calcium, no magnesium, no phenol red | ThermoFisher Scientific, Gibco™ | 14175095 |
| UltraPure™ 0.5M EDTA, pH 8.0 | ThermoFisher Scientific, Invitrogen™ | 15575020 |
| DTT, Crystalline powder | Millipore Sigma | 10197777001 |
| Fetal Bovine Serum (Heat Inactivated) | Millipore Sigma | F4135-500ML |
| RPMI-1640 Medium | Millipore Sigma | R8758 |
| Liberase™ TL Research Grade | Millipore Sigma | 05401020001 |
| DNase I, RNase-free (1 U/µL) Share | ThermoFisher Scientific | EN0521 |

bioRxiv preprint doi: https://doi.org/10.64898/2026.05.15.725500; this version posted May 16, 2026. The copyright holder for this preprint (which was not certified by peer review) is the author/funder, who has granted bioRxiv a license to display the preprint in perpetuity. It is made available under a CC-BY 4.0 International license.

| | | |
|---|---|---|
| Cytiva Percoll™ Centrifugation Media | Cytiva | 17089102 |
| TRIzol™ | ThermoFisher Scientific, Invitrogen™ | 15596026 |
| HyClone™ HyPure Water, Molecular Biology Grade | Cytiva | SH30538.FS |
| Phosphate-buffered saline, no calcium or magnesium (PBS) | ThermoFisher Scientific, Gibco™ | 14190144 |

**Table S16: Primers**

| **Primer** | **Sequence** |
|---|---|
| β-Actin_fwd | GGCTGTATTCCCCTCCATCG |
| β-Actin_rev | CCAGTTGGTAACAATGCCATGT |
| B2m_fwd | CTCACGCCACCCACCGGAGAAT |
| B2m_rev | GCCAGGATATAGAAAGACCAGTCCTTGCTG |
| Bmal1_Exon5_fwd | GCCCACAGTCAGATTGAAAAGAGGCG |
| Bmal1_Exon5_rev | TGCTGAACAGCCATCCTTAGCACG |
| Ddx60_fwd | TGTAGGGCGTGCCTAGGTCAGAG |
| Ddx60_rev | TCCCTGCCACTCACCACTCAGAC |
| Erap1_fwd | GTAGAAATCATAGCTAGCCGGCCCACC |
| Erap1_rev | CTTCAGACAGCATCTCTCCAGCTCCTC |
| Ifih1_fwd | AGAGCGGTCCCGGGTCTGTG |
| Ifih1_rev | TGTGTCCCCGCCTGAACTGC |
| Ifit1_fwd | AAGGGCTCTGCTACAAGCAA |
| Ifit1_rev | ATCTCAAATGTGGGCCTCAG |
| Irf1_Cloning_fwd1 | GTACGGGAGGTATTGGACA |
| Irf1_Cloning_rev1 | AGTACTATCGATTCACACAAAAA |
| Irf1_Cloning_fwd2 | CACACAGCCTGAAACTCC |
| Irf1_Cloning_rev2 | TCAAACATGTGTGTGTACAGTG |

bioRxiv preprint doi: https://doi.org/10.64898/2026.05.15.725500; this version posted May 16, 2026. The copyright holder for this preprint (which was not certified by peer review) is the author/funder, who has granted bioRxiv a license to display the preprint in perpetuity. It is made available under a CC-BY 4.0 International license.

| | |
|---|---|
| Irf1_Cloning_fwd3 | GATTCAAACCCAACCCAC |
| Irf1_Cloning_rev3 | GGAATGTGATTTGTGAGCTT |
| Irf1_Cloning_fwd4 | ACTCCATGCCTCCAATTC |
| Irf1_Cloning_rev4 | GCTAAGCTCAAGGCTAGAAA |
| Irf1_Cloning_fwd5 | CTCAGGTATGTGAGACTTTGTC |
| Irf1_Cloning_rev5 | AAGGGATACCTGAGGAATGA |
| Irf1_Cloning_fwd6 | CCAGGGTACTGCGAGTTAT |
| Irf1_Cloning_rev6 | CAGGAGAAGTACAGGGGG |
| Irf1_Cloning_fwd7 | AGGCCACACAGCTTATAGAT |
| Irf1_Cloning_rev7 | TCTACAATGTTTTTACTTTCAG |
| Irf1_DNA_Binding_Domain_Segment6_fwd | AGAAAGTCCAAGTCCAGCCGAGACACTAAG |
| Irf1_DNA_Binding_Domain_Segment6_rev | GTAACTGCTGTGGTCATCAGGTAGGG |
| Irf1_Ebox1_fwd | CACTGTACACACACATGTTTGAG |
| Irf1_Ebox1_rev | CGTGGTGAGGCAGCTTTG |
| Irf1_Ebox2_fwd | TCCCACTGGAAGTTCCAGC |
| Irf1_Ebox2_rev | CGTGGAGGCAACAAGGAAAT |
| Irf1_Exon1_fwd | GATTTCTTCGCGGCGCC |
| Irf1_Exon1_rev | CTGGCCACCTCGGCT |
| Irf1_Exon10_fwd | GCATAGTCCCACTGCAAACA |
| Irf1_Exon10_rev | GCCTCTGCCTTACACCTCAG |
| Irf2_Exon9_fwd | GGATAGCTGTCCGATGCC |
| Irf2_Exon9_rev | GCCTGGGTGATATCAGATGTC |
| Irf3_Exon8_fwd | TGTCCTGCCTCCTTCAGG |
| Irf3_Exon8_rev | GGTAGGCCTTGTACTGGTCA |
| Irf4_Exon9_fwd | GAGTGGGTTTCCACTTGAAGA |
| Irf4_Exon9_rev | CAGTTATAAGCCAGGGCACAG |

bioRxiv preprint doi: https://doi.org/10.64898/2026.05.15.725500; this version posted May 16, 2026. The copyright holder for this preprint (which was not certified by peer review) is the author/funder, who has granted bioRxiv a license to display the preprint in perpetuity. It is made available under a CC-BY 4.0 International license.

| | |
|---|---|
| Irf5_Exon9_fwd | CAGCAGCAATTGCAGCC |
| Irf5_Exon9_rev | GCTATATCAGTCTACGTGGCTG |
| Irf6_Exon8_fwd | CGTGTCCTCTTCTCTCCTCTC |
| Irf6_Exon8_rev | CCTCAGGGGATGATACCTTGATAAG |
| Irf7_Exon10_fwd | GTCCTTTCCACAGCTGGAG |
| Irf7_Exon10_rev | CCAGGTCCATGAGGAAGTGT |
| Irf8_Exon7_fwd | GTACCTCACACCAGAGACCC |
| Irf8_Exon7_rev | GTCAGAGGGCTCCACATC |
| Irf9_Exon9_fwd | GAAGACTCGCCTACGCTGTG |
| Irf9_Exon9_rev | GGGTTCCTGCTGGCAGTATT |
| Oas2_Cloning_fwd1 | ACCAGGAAAGGGAGAGGTGT |
| Oas2_Cloning_rev1 | CCAGTACTTGCTGGGGAGTC |
| Oas2_Cloning_fwd2 | CGTCTTGGACCAGGAAGAAA |
| Oas2_Cloning_rev2 | CAGAGGGTGTGGAGAGGAAG |
| Oas2_Cloning_fwd3 | GACCAGCCAGATGAATAGCC |
| Oas2_Cloning_rev3 | AGGCCCAGGACTTGAGCTAC |
| Oas2_Exon_fwd | ACCAGGAAAGGGAGAGGTGT |
| Oas2_Exon_rev | CCAGTACTTGCTGGGGAGTC |
| Oas2_ISRE_fwd | GGACCCAGGTTATAGTGTCTCCATCA |
| Oas2_ISRE_rev | GGAACACGCCGTCACTGGTT |
| Oas3_Exon_fwd | CTGAGAGTACCGGTCAACATGGACC |
| Oas3_Exon_rev | CGGGCTCTGTGCTGCTGTAGG |
| Per2_fwd | TGCCCTGAGTTCTCCAGTTT |
| Per2_rev | CAGTGATCTGAGGACCAGCA |
| Rig-I_fwd | CCTATGGCCAGCCGGCTAGG |
| Rig-I_rev | GCTGAGGATGTAGGTGGGGTCCAG |

bioRxiv preprint doi: https://doi.org/10.64898/2026.05.15.725500; this version posted May 16, 2026. The copyright holder for this preprint (which was not certified by peer review) is the author/funder, who has granted bioRxiv a license to display the preprint in perpetuity. It is made available under a CC-BY 4.0 International license.

| | |
|---|---|
| Stat1_fwd | TGATGTCTCGTTTGCGACCA |
| Stat1_rev | ACCTGGAGATTACGCTTGCT |
| Stat2_fwd | GCAGTGGGAGATGTTGCAGA |
| Stat2_rev | CCAAATGCTGTCGAACGTCC |
| Tapbpl_fwd | CACCTGGGGATTTCTTGGGTGCG |
| Tapbpl_rev | CCTCACTTAGCACCTGTGGTCTCCG |
| Tlr3_fwd | GAGGAAGAAGGAGTTAATCCCGAGCTC |
| Tlr3_rev | GGAGGTCCTGCCTCAAAAGCAGG |

bioRxiv preprint doi: https://doi.org/10.64898/2026.05.15.725500; this version posted May 16, 2026. The copyright holder for this preprint (which was not certified by peer review) is the author/funder, who has granted bioRxiv a license to display the preprint in perpetuity. It is made available under a CC-BY 4.0 International license.

## Supplemental References


38. Y. Wang, J. K. Pfeiffer, Emergence of a large-plaque variant in mice infected with coxsackievirus B3. *MBio* **7**, 10.1128/mbio. 00119-00116 (2016).

39. C. M. Robinson, Y. Wang, J. K. Pfeiffer, Sex-dependent intestinal replication of an enteric virus. *Journal of virology* **91**, 10.1128/jvi. 02101-02116 (2017).

40. R. Parsons, N. Garner, H. Oster, O. Rawashdeh, CircaCompare: a method to estimate and statistically support differences in mesor, amplitude
